# Sharing Emotions Contributes to Regulating Collaborative Intentions in Group Problem-Solving

**DOI:** 10.3389/fpsyg.2020.01160

**Published:** 2020-06-16

**Authors:** Sunny Avry, Gaëlle Molinari, Mireille Bétrancourt, Guillaume Chanel

**Affiliations:** ^1^SIMS Laboratory, Department of Computer Science, University of Geneva, Geneva, Switzerland; ^2^TECFA Laboratory, Department of Psychology and Education Science, University of Geneva, Geneva, Switzerland

**Keywords:** emotion sharing, collaborative learning, collaborative problem solving, socio-cognitive processes, socio-relational processes, socio-epistemic processes, collaborative acts

## Abstract

Collaborative problem-solving has been gaining attention as more and more students and employees work together all around the world to find solutions to complex problems. This trend goes hand in hand with a growing interest in the role of affective processes in learning and problem-solving fields. However, the comprehension of real-time dynamics between emotional sharing and collaborative exchanges (what we propose to call “collaborative act”) still needs to be deepened. The challenge is especially on understanding the interplay between real-time changes in epistemic and relational dimensions. In this study, we propose to explore this question in dyadic creative problem-solving. Eleven pairs of participants used an argument graph tool to co-create a slogan against violence at school. The tool was used to write down slogans and build a joint map of the group argumentation. During the collaboration, they had access to an emotion awareness tool, allowing them to share emotional labels in real time. An indicator of real-time use was computed to track ongoing changes in collaborative acts during collaboration. Then, using both inferential and descriptive statistics, we first investigated whether emotional sharing induces real-time adaptation of both emitter’s and receiver’s collaborative acts. Second, we looked at privileged relationships between emitter’s collaborative acts, emitter’s emotion sharing, and receiver’s collaborative acts. The preliminary results obtained (1) confirm that emotional sharing regulates emitter’s and receiver’s collaborative acts and (2) strongly suggest that specific emotions mark specific patterns of collaboration in different collaborative phases, implying both the epistemic and the relational spaces of collaboration. These results highlight the value of studying emotional sharing for a deeper comprehension of the factors regulating collaborative problem-solving. Perspectives in educational psychology and computer science are considered, with the will to understand and promote better self- and co-regulation of collaborative problem-solving through emotional sharing.

## Introduction

Problems are omnipresent throughout daily life. Getting into a given place in an unknown city or sending a rocket into space both imply problem-solving, at different levels of complexity. As the problems we encounter tend to become more and more complex in today’s world, they often require inputs from others. Therefore, collaboration is increasing all around the world as more and more people work together to solve non-routine problems and lead innovation (Fiore et al., 2017; Borge et al., 2018; Graesser et al., 2018). In academic settings also, learners are regularly required to solve problems together. However, what makes a collaboration successful is still unclear since, as Barron (2003) raised, putting problem-solvers together, as smart as they are, is not a guarantee of better success. On the contrary, group success heavily depends on the quality of real-time interaction (Barron, 2003; Borge et al., 2018), especially the responsiveness to the other group members. In such a context, the affective states shared in collaborative settings could play a crucial role in the collaborators’ mutual adaptation, i.e., socio-metacognition (Borge et al., 2019). These adaptive changes could affect collaborative intentions (what we propose to call “collaborative acts”) dedicated to both solving the problem and managing the relationship between problem-solvers. In this study, we explore this question in analyzing a computer-supported collaborative problem-solving task, where real-time emotion sharing was recorded during the collaboration.

### Collaborative Acts Instantiate Collaborative Processes

Collaborative problem-solving can be defined as the “capacity of an individual to effectively engage in a process whereby two or more agents attempt to solve a problem by sharing the understanding and effort required to come to a solution and pooling their knowledge, skills and efforts to reach that solution” (Fiore et al., 2017, p.6). In recent years, the idea that collaboration involves two interrelated spaces, namely, epistemic and relational, has been gaining ground (Roschelle and Teasley, 1995; Barron, 2003; Andriessen et al., 2011). The epistemic space globally refers to the processing of information dedicated to solving the problem, while the relational space is related to the peer relationships. Therefore, throughout the collaboration, problem-solvers mobilize processes dedicated to managing the epistemic and the relational spaces. For example, in the epistemic space, a possible solution can be shared with the others (socio-cognitive process) to address the problem. At the same time, in the relational space, one can display responsiveness to other participants or some marks of solidarity to re-engage a partner that undergoes a lack of motivation (socio-relational process).

One question that arises from the above description is how the different processes take form in the discourse during collaboration. One proposition is that collaboration involves different collaborative processes (e.g., information management and argumentation management). These processes emerge iteratively and incrementally through communicative exchanges. These communicative exchanges can be considered as speech acts (Austin, 1975), i.e., they involve an intention from the speaker and an effect on the listener (e.g., provide information, clarify an idea, ask for help, and encourage). These speech acts are intended to produce perlocutionary effects, i.e., consequences on feelings, thoughts, and actions of others (Sbisà, 2009). In the framework of collaboration, we propose to call them collaborative acts as they represent a sub-category of speech acts involving collaborative intentions (in contrast with competitive intentions, for example). These collaborative acts build socio-cognitive and socio-relational processes and shape the course of collaboration in feeding mutual models (Dillenbourg et al., 2016). Mutual modeling can relate to knowledge (Sangin et al., 2007). For example, collaborative acts dedicated to asking information could induce information sharing and update each other’s knowledge model of the task (i.e., online task-specific knowledge; see Efklides, 2011), the self, and the partner (Sangin et al., 2007). If knowledge models relate to the epistemic space, similar models are also posited about the relational space (e.g., updates of mutual attitudes, dispositions, and beliefs) (Dillenbourg et al., 2016), even if no clear empirical evidence of such relational models is yet available in the literature.

A sequence of collaborative acts with the same perlocutionary effect can, therefore, be associated with a given socio-epistemic or socio-relational process. For example, a series of collaborative acts dedicated to collecting and evaluating an argument or critically assessing a proposition may fall within the reaching consensus process (see Meier et al., 2007). The understanding of how, and under which conditions, a sequence of collaborative acts can forge successful or unsuccessful collaborative patterns is an essential question in collaborative problem-solving. To this end, coding and categorizing collaborative acts into well-defined collaborative processes (e.g., socio-epistemic and socio-relational) can contribute to exploring the course of collaboration more finely. Several frameworks have been developed in the (computer-supported) collaborative learning field to extract meaning from collaborative exchanges occurring during collaboration (e.g., Bales, 1950; Baker et al., 2007; Hughes et al., 2007; Meier et al., 2007; Noroozi et al., 2012). The purpose of these frameworks is twofold. First, they help to classify speech utterances and group them into meaningful collaborative processes. Second, they give an overview of what is happening in the collaboration. By putting side by side group outcomes and collaborative processes profiles, it could thus be possible to get an idea about what are the dimensions of a good collaboration. For example, Kahrimanis et al. (2009) characterized good collaboration along seven dimensions. Some dimensions refer to socio-relational processes (e.g., cooperative orientation), while some others to socio-epistemic processes (e.g., sustaining mutual understanding and knowledge exchange). Some of these dimensions correlate positively with the mental representation of good collaboration held by the participants (Meier et al., 2007).

### Emotional Expression Regulates Social Interactions

If cognition and emotion have historically been opposed, with emotion being thought of as impeding cognition (Huntsinger and Schnall, 2013), the role of emotion in intraindividual cognition is now well documented (e.g., Spering et al., 2005; Isen and Labroo, 2012; Fredrickson, 2013; Lerner et al., 2015; George and Dane, 2016). For example, evidence shows that emotions trigger prototypical cognitive dispositions to evaluate events in a way that modulates the interpretation of subsequent situations (Lerner et al., 2015). For example, anger tends to make negative events more predictable and under control (Van Doorn et al., 2015). In return, that could eventually lead to underestimating risk (Lerner et al., 2015) and may serve a social distancing function toward people and situations (Fischer and Manstead, 2008). In academic settings also, intraindividual emotions are now considered critical for students’ learning and problem-solving, especially academic achievement (Pekrun, 2006; Pekrun and Linnenbrink-Garcia, 2012; Avry et al., 2020).

Interest in the social functions (beyond survival and reproduction) of emotions has emerged more lately (Keltner and Haidt, 1999; Morris and Keltner, 2000). However, emotions are nowadays also thought to have a significant role in social decision making. Morris and Keltner (2000) emphasize the social role of emotion as the consequences of emotion that occur between people who are observing and responding to each other’s emotions rather than as the consequences within one individual. Indeed emotions are often elicited by others and expressed to influence others (Van Kleef et al., 2016). A crucial question thus concerns the function of emotional expressions in group settings, from both the emitter’s and the receiver’s points of view. This is of great importance for the understanding of the role of emotions in collaboration since we know that emotions intervene in the coordination of group efforts to achieve shared goals (Van Kleef et al., 2016).

First, one can consider how the emitter uses emotions to convey messages to others. People have a natural tendency to share their emotions with others (Rimé, 2009). Emotional expressions allow people to regulate emotional (e.g., seeking for consolation), motivational (e.g., need for encouragement), and epistemic (e.g., looking for advice and solutions) aspects of interaction [see Rimé (2007) for a comprehensive study of the different motives for socially sharing an emotion]. However, depending on the group context, the willingness to share emotions is different (e.g., contrary to work meetings, group support meetings could promote the sharing of more negative emotions) (Van Kleef et al., 2016). In addition, Andriessen et al. (2011) also emphasized the need to consider collaborative learning as a continuous cycle of tensions and relaxations at both epistemic and relational levels, which pervade the group through emotional expressions and contagion. Thus, different exchanges that occur in the collaboration each embed an idiosyncratic potential to increase or alleviate group tensions. For example, tensions may arise at a socio-relational level from touches of sarcasm or personal attacks, while irrelevancy claims, tough questions, or deep reflection may provoke tensions at a socio-cognitive level.

Second, one can consider how the receiver uses emotional expressions to infer information (Van Kleef and Fischer, 2016). For example, in collaborative problem-solving, one can consider the kind of information that is inferred through emotional expressions. Some lines of response can be proposed to understand this issue. Perceived emotional expressions could serve as a social warning, inducing the observer to focus on the emotional state highlighted by the emotional expression. In this line, Van Kleef et al. (2016) outline that emotional expressions help to prove the expresser’s interpretation of a situation. For example, during a collaborative problem-solving task, if a collaborator begins to frown, the others should be induced to put attention on it as frowning is likely to be interpreted as a negative effect in that context (see affective cognition; Ong et al., 2015). Therefore, collaborators can infer, from the highlighted emotional state, the causes and the future consequences of that emotional state and adapt themselves accordingly, considering the context that led to its emergence (Van Kleef et al., 2010). In this way, emotional expressions can help to infer not only the other’s beliefs, social intentions, and relationship orientations (e.g., dominant or submissive and receptive or indifferent) (Keltner and Haidt, 1999) but also the degree of cooperativeness, the competence, and the personality, among others (Van Doorn et al., 2015).

Therefore, in collaborative settings, emotional expressions could, at specific points of time, (1) from the emitter’s point of view, help to draw the other’s attention implicitly and explicitly on socio-relational and socio-epistemic matters and (2) from the receiver’s point of view, focus on other’s emotional state to make inferences about the emitter, reduce ambiguity, and adapt to the emitter’s needs.

### Research Questions and Hypotheses

Various areas of research have linked cognitive processes to emotions (Eastwood et al., 2001; Spering et al., 2005; D’Mello and Graesser, 2012; Lerner et al., 2015). For example, D’Mello and Graesser (2012) explain how emotions and the cognitive processing of information are intertwined in individual complex learning. In their model, specific emotional (or cognitivo-affective) states go hand in hand with specific cognitive states (flow with equilibrium, confusion with disequilibrium, frustration with stuck, and disengagement with boredom). As cognitive reasoning is also conveyed through communicative exchanges in collaboration, a question that can be asked is whether similar findings can be found between emotion sharing and collaborative acts. Furthermore, as outlined in the previous section, emotional sharing is not only related to socio-epistemic matters. Literature also shows that socio-emotional matters are a significant concern in social interaction (Rimé, 2007). In addition, Pekrun and Linnenbrink-Garcia (2012) distinguish several types of emotions occurring in individual academic settings that are related to specific focuses of learning, such as achievement emotions (related to the achievement of activity and outcomes; e.g., the frustration of not succeeding), epistemic emotions (related to the learner’s cognitive processing of information; e.g., the confusion of not understanding a problem), or social emotions (related to the relationship with others; e.g., the gratitude toward a peer). This categorization suggests that emotional sharing could also be related to different collaborative focuses, primarily epistemic and relational, that could be shared preferentially in different phases of collaboration (e.g., when problem-solvers make acquaintance or try to find new ideas to solve the problem).

We proposed above that the emitter would use emotions to draw the receiver’s attention regarding important emotional, motivational, or cognitive matters, which would be intended to induce perlocutionary effects from the receiver. Consequently, emotional expressions would lead the receiver to make inferences about the emitter’s needs, which would induce adaptive effects in return. These adaptive changes are posited to occur in real time, before emotional sharing for the emitter and after emotional sharing for the receiver.

Therefore, the first questioning that drives this study is whether emotion sharing modulates collaborative acts (RQ1). First, we assume that real-time changes of specific collaborative acts by the emitter precede specific emotional sharing by the emitter (H1a). From an operational point of view, specific collaborative acts should be subject to a significant increase (respectively, decrease) preceding specific emotional sharing, compared to when no emotion is shared. For example, if the emitter has a strong positive opinion about a possible solution to solve the task, he/she should be more likely to draw the receiver’s attention by sharing an emotion of interest. Second, we assume that real-time changes of some specific collaborative acts by the receiver follow specific emotional sharing by the emitter (H1b). For example, if the emitter shares an emotion of interest, the receiver should adapt his/her collaborative acts accordingly.

A second issue that arises from the literature concerns the relationships between the sharing of some emotions and some patterns of collaboration. Therefore, the second questioning is whether specific patterns of collaboration can be highlighted, considering the triad emitter’s collaborative acts, emotional sharing, and receiver’s collaborative acts, and if these patterns occur preferentially in specific collaborative phases (RQ2). We assume that specific triads relate more specifically to dealing with specific epistemic or relational matters (H2a). We also assume that some triads occur preferentially in specific collaboration phases (H2b).

## Materials and Methods

### Participants

The analysis was performed on data provided from a sample of 22 participants (12 women and 10 men, *M* = 23.9 years; SD = 7.45) taken from the freely accessible EATMINT database^1^ (Chanel et al., 2013), regrouping multi-modal and multi-user data of affect and social behaviors recorded during a computer-supported creative problem-solving collaboration (Molinari et al., 2013), such as physiological signals (electrocardiogram, electrodermal activity, blood volume pulse, respiration, and skin temperature), behaviors (eye movements, facial expressions, and software action logs), and discourse (speech signals and transcripts).

### Procedure

**FIGURE 1:**
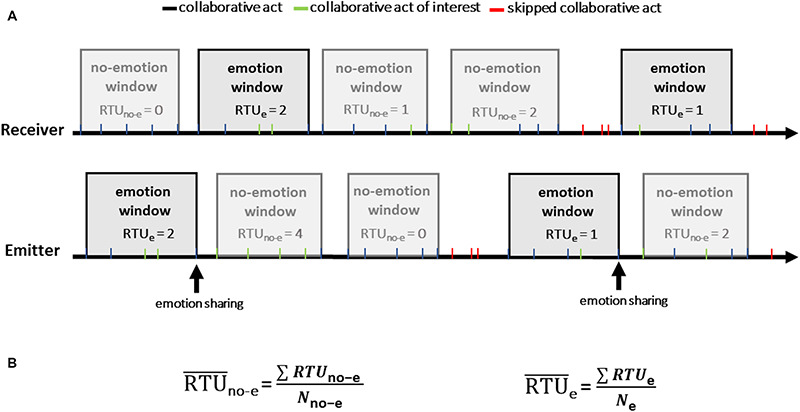
Example showing the collaborative acts divided into windows of five consecutive collaborative acts. In **(A)**, the emotion windows of the emitter include five collaborative acts preceding the sharing of a given emotion by the same emitter. The emotion windows of the receiver include five collaborative acts following the sharing of a given emotion by the emitter. In this case, the collaborative acts of interest are those emitted by the receiver. In **(B)**, ${\bar{\text{RTU}}}_{\mathrm{no}-e}$ is equal to the sum of the RTU_*no–e*_ divided by the number of non-emotion windows (*N*_*no–e*_). ${\bar{\text{RTU}}}_{\text{e}}$ is equal to the sum of the RTU_*e*_ divided by the number of emotion windows (*N*_*e*_).

Eleven dyads of participants using networked computers were involved working together in the DREW software (Jaillon et al., 2002), a collaborative environment that includes an argument graph tool which allow collaborators to build a joint map of their argumentation. The participants could communicate through microphone headsets, and their verbal exchanges were recorded. They did not see each other. The participants were asked to use the argument graph tool to create a slogan against violence at school collaboratively (Figure 1, left). The group collaboration lasted for about 36 min on average. It was divided into three main phases. The participants should spend two-fifths of the time in phase 1 where they should produce as many slogan ideas as possible, two-fifths of the time in phase 2 where they should debate with each other and agree on three slogans, and one-fifth of the time in the last phase where they should choose the best slogan.

The dyad members were also provided with a tool which allow them to share, in real time, verbal labels of their emotions through an emotion awareness tool (Figure 1, right). They could choose among 10 positive (delighted, focused, interested, satisfied, empathic, confident, amused, relaxed, grateful, and relieved) and 10 negative (stressed, annoyed, surprised, disappointed, envious, anxious, dissatisfied, confused, frustrated, and bored) emotions by clicking on them. The emotions available in the emotion awareness tool were chosen based on a pre-test which aimed at identifying the most frequent emotions used during a collaborative task. Once the participants selected an emotion, it was automatically displayed to them (green area in Figure 1) as well as their partner (blue area in Figure 1). The participants were instructed that they were free to self-report their emotions at any time they wanted during the collaboration (for a complete description, see Molinari et al., 2013). In addition, they were prompted with a pop-up window to share their emotions at the beginning of the interaction and every 5 min during the collaboration.

### Analyses

#### Speech Coding

A coding scheme was developed to code speech utterances into different collaborative acts during the collaboration. It was composed of 27 collaborative acts grouped into six collaborative processes (Table 1). In this coding scheme, a collaborative process is composed of one or several collaborative acts that have specific perlocutionary effects. For instance, collaborative acts aiming at complimenting or encouraging the collaborators are combined into a collaborative process called *relationship management*. The Rainbow model (Baker et al., 2007) was chosen as a working basis for the creation of the coding scheme. This model was initially developed for coding speech utterances from chat interactions in seven broad collaborative processes: outside activity, social relation, interaction management, task management, opinions, argumentation, and broaden and deepen. Only outside activity, social relation, and interaction management categories were retained, while information, argumentation management, and tool management categories were added afterward. Some categories were refined in sub-categories based on other coding schemes (Bales, 1950; Hughes et al., 2007; Meier et al., 2007; Noroozi et al., 2012). The final coding scheme (Table 1) obtained provides a functional classification (each collaborative act refers to a particular collaborative process) which aims at covering the largest possible types of collaborative acts that occur in collaborative problem-solving. Emphasis was also put on both socio-relational and socio-epistemic processes. For each dyad, the whole verbal content of interactions was transcribed with the ELAN software (Sloetjes and Wittenburg, 2008). Pauses and turns taking served as a basis for segmenting the verbal interaction into speech utterances. When appropriate, each speech utterance was coded as a collaborative act. Speech utterances related to problem-solving but not falling within any other collaborative act category were coded as *other.* Speech utterances unrelated with problem-solving were coded as *outside activity*. The speech content was categorized by a first expert coder, whereas a second naive coder with no prior experience on collaborative processes coding scheme literature was in charge of 10 dyads. The inter-coder reliability for the 27 collaborative acts on these 10 dyads was calculated as the Cohen’s kappa coefficient and was equal to 0.52 (moderate agreement; Viera and Garrett, 2005). The inter-coder reliability for the six collaborative processes was equal to 0.61 (substantial agreement). The categorization carried out by the first expert coder was used as part of this study.

**TABLE 1:** Coding scheme developed to code speech utterances into collaborative acts.

Collaborative process	Definition of the collaborative process	Collaborative act	Definition of the collaborative act
Relationship	Management of relational	Display solidarity	Compliment or encourage partner or group
management	aspects of collaboration	Display hostility	Depreciate or disregard partner or group
		Relax atmosphere	Improve atmosphere or alleviate tensions (humor, laughs, teasing)
		Use social convention	Greet, display courtesy, introduce each other
Interaction	Management of group	Check reception	Initiate or check contact with partner
management	interaction	Check comprehension	Check comprehension of what partner previously said
		Display active listening	Communicate attentive listening of partner
		Display reflection	Communicate moment of reflection to partner
		Coordinate teamwork	Manage role distribution
		Accept coordination	Accept group coordination
		Refuse coordination	Object to group coordination
Information	Management of group	Give task information	Give information that can help to solve the problem or remind the rules or task constraints
management	information	Give explanation	Clarify/elaborate one’s own thinking
		Elicit task information	Ask information that can help to solve the task or reminding the rules or task constraints
		Give self information	Give an information about one’s own knowledge or thinking
		Elicit partner information	Ask information about partner’s knowledge or thinking
		Give recall	Repeat former information
		Elicit recall	Ask again former information
Argumentation	Management of group	Give proposition	Propose idea to resolve the task
management	argumentation	Give positive opinion	Support proposed idea
		Give negative opinion	Contradict proposed idea
		Elicit proposition	Elicit new idea from partner
		Elicit opinion	Elicit partner’s opinion
		Agree	Agree with proposed idea
		Incorporate	Enriching proposed idea
Task management	Management of collaborative task	Manage task	Manage task progress, what has been done, and what is still to be done
Tool management	Management of collaborative tool	Manage tool	Manage collaborative tool usage
Other	Other	Other	Communication related to problem-solving task but not falling within any previous category
Outside activity	Outside activity	Outside activity	Communication unrelated with problem-solving task

#### Computation of Real-Time Collaborative Acts Use

**FIGURE 2:**
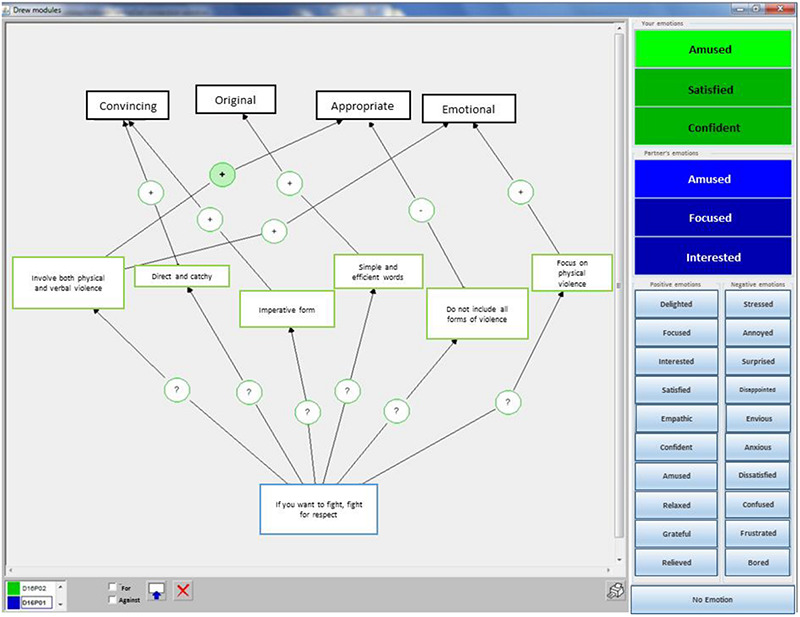
The Drew interface coupled with an emotion awareness tool for sharing verbal labels of emotions in real-time during the collaborative problem-solving task.

In order to measure the real-time impact of emotional sharing on collaborative acts, we computed the number of collaborative acts of a given type produced by the emitter before one’s emotion sharing and by the receiver after the emitter’s emotional sharing (Figure 2A). Emotion windows (i.e., the *n* collaborative acts preceding or following the sharing of a given emotion) were first created (Figure 2A). Initially, different window sizes (5, 10, and 15 collaborative acts) were tested. The windows of five collaborative acts were retained as the effect of emotion sharing was the strongest for this size.

To determine the beginning of an *emotion window*, the shared emotion was associated with the temporally closer collaborative act. *No-emotion windows* were then created for the remaining collaborative acts. When the number of collaborative acts was inferior to five (e.g., between two *emotion windows*), a window was not created (cf. skipped collaborative acts in Figure 2A). Furthermore, when two *emotion windows* overlapped, the second one was skipped to avoid dependencies between emotional windows. The creation of windows gave the possibility to focus on collaborative act changes in real time (five acts lasted for 13.2 s on average).

After the creation of windows, real-time use (RTU), defined as the number of occurrences of a given collaborative act in the window of five collaborative acts, was then computed for each window. For example, if the collaborative act *give proposition* occurred three times among five consecutive collaborative acts, the RTU for this given collaborative process in this sample was three. Then, the different RTU scores for the *emotion* and *no-emotion windows* were averaged (Figure 2B). Therefore, as part of a given shared emotion, each participant was associated, for each type of collaborative act, with a pair of two dependent scores, a score representing the averaged RTU for the emotion windows (${\bar{\text{RTU}}}_{\text{e}}$) and a score representing the averaged RTU for the no-emotion windows (${\bar{\text{RTU}}}_{\mathrm{no}-e}$) (Figure 2B). This process of creation of windows and computation of the RTUs was carried out for all the shared emotions considered in this study.

## Results

### Overall Descriptive Statistics

The whole sample contained 5,141 collaborative acts that the participants have initiated during the collaboration (467.36 ± 194.18 collaborative acts per dyad). The number of collaborative acts per participant ranged from 63 to 380. A collaborative act lasted 2.64 ± 2.80 s and the cumulated duration of collaborative acts was on average 20 min 3 s ± 8 min 43 s. Supplementary Table A1 reports the number of collaborative acts in each category in the whole sample as well as the percentage of each act relative to the total number of acts.

The whole sample studied contained 262 (232 positive and 30 negative) shared emotions (23.90 ± 9.78 emotions per dyad). The number of shared emotions per participant ranged from 5 to 29. On average, the absolute difference in the number of shared emotions by each participant within the same dyad was 4.63 ± 4.20, ranging from 0 (i.e., both partners shared the same number of emotions) to 13. The absolute difference in the number of negative shared emotions by each participant within the same dyad was 1.81 ± 1.53, ranging from 0 to 4. The absolute difference in the number of positive shared emotions by each participant within the same dyad was 2.91 ± 3.17, ranging from 0 to 9. Supplementary Table A2 reports the number of emotions in each category in the whole sample as well as the percentage of each emotion relative to the total number of emotions. An emotion was released every 1 min 43 ± 38 s on average, ranging from 57 s to 2 min 45 s.

Given that all the emotions were not shared by all the participants (see Supplementary Table A2), analyses were carried out when emotion was shared at least once in at least 10 participants to preserve the statistical power. Under this constraint, the following emotions were retained: *interest*, *focused*, *amused*, *relaxed*, *satisfied*, and *delighted*. These six emotions were not shared equally in each collaborative phase (Table 2). Similarly, because some collaborative acts were shared very rarely, only the collaborative acts used more than 4% of the time were retained, i.e., *relax atmosphere*, *display reflection*, *accept coordination*, *give task information*, *give self information*, *give proposition*, *give positive opinion*, *agree*, *manage task*, and *manage tool*. *Relax atmosphere* relates to the relational space and the other collaborative acts to the epistemic space.

**TABLE 2:** Numbers of emotion sharing according to the different phases of collaboration.

Phase	Interested	Focused	Amused	Relaxed	Satisfied	Delighted
Produce as many slogan ideas as possible	22	24	22	9	10	8
Debate with each other and agree on three slogans	14	20	6	4	14	1
Choose the best slogan	3	8	6	6	16	11

### Randomization Tests

For each couple of shared emotion and collaborative act, a randomization test was carried out to test a significant difference between ${\bar{\text{RTU}}}_{\mathrm{no}-e}$ and ${\bar{\text{RTU}}}_{\text{e}}$ across the samples. The randomization test allows for testing the relationship equality of means when one cannot assume the normality of the test statistic. First, the true difference of means was computed in the sample of size N. Second, the set of ${\bar{\text{RTU}}}_{\text{e}}$ and the set of ${\bar{\text{RTU}}}_{\mathrm{no}-e}$ were shuffled together, and a random difference of means ($\bar{r\,a\,n\,d.d\,i\,f\,f}$) was computed in the same way. This operation was repeated 9,999 times, resulting in a sampling distribution of random differences. The *p*-value was computed as the proportion of permuted datasets which produced a mean difference at least as extreme as the true difference (two-tailed testing) using the following formula:

$$p-\mathrm{value}=\frac{\sum_{1}^{10,000}\left(|\bar{\mathrm{rand}.\mathrm{diff}}|\geq |\bar{\mathrm{true}.\mathrm{diff}}|\right)}{N}$$

Third, the set of *p*-values obtained for each shared emotion obtained has been corrected for multiple comparisons with the Benjamini–Hochberg procedure.

### Effect of Emotion Sharing on Real-Time Collaborative Acts Use

A series of randomization tests was conducted to test (1) the effects of emotion sharing on the $\bar{\text{RTU}}\,\text{s}$ of the previous emitter’s collaborative acts and (2) the effects of emotion sharing on the $\bar{\text{RTU}}\,\text{s}$ of the following receiver’s collaborative acts. Comprehensive descriptive results are reported in Tables 3, 4.

**TABLE 3:** Comprehensive results for the emitter for each couple of shared emotion and collaborative act.

Collaborative acts	Interested (*N* = 19)				Focused (*N* = 21)				Amused (*N* = 18)				Relaxed (*N* = 12)				Satisfied (*N* = 16)				Delighted (*N* = 11)			
	*p*	*M−* (SD)	*M*+ (SD)	Variation *%*	*p*	*M−* (SD)	*M*+ (SD)	Variation *%*	*p*	*M−* (SD)	*M*+ (SD)	Variation *%*	*p*	*M−* (SD)	*M*+ (SD)	Variation *%*	*p*	*M−* (SD)	*M*+ (SD)	Variation *%*	*p*	*M−* (SD)	*M*+ (SD)	Variation *%*
Agree	0.89	0.51 (0.26)	0.54 (0.67)	+5, 88	0.86	0.51 (0.25)	0.46 (0.62)	*−*9, 80	0.81	0.46 (0.13)	0.61 (0.85)	+32, 61	1.00	0.48 (0.08)	0.44 (0.68)	*−*8, 33	0.91	0.52 (0.25)	0.56 (0.88)	+7, 69	0.87	0.44 (0.10)	0.53 (0.70)	+20, 45
Accept coordination	0.51	0.46 (0.22)	0.31 (0.39)	*−*32, 61	0.69	0.43 (0.22)	0.54 (0.48)	+23, 26	0.39	0.42 (0.22)	0.22 (0.36)	*−*47, 62	0.85	0.34 (0.11)	0.56 (0.53)	+64, 71	0.20	0.41 (0.18)	0.22 (0.35)	*−*46, 34	0.74	0.43 (0.19)	0.24 (0.56)	*−*44, 19
Give positive opinion	0.89	0.67 (0.25)	0.57 (0.74)	-14, 93	**0.02**	0.69 (0.25)	0.32 (0.41)	-53, 62	0.88	0.67 (0.25)	0.62 (0.71)	-7, 46	0.87	0.69 (0.28)	0.56 (0.85)	-18, 84	0.91	0.66 (0.26)	0.56 (0.55)	-15, 15	**0.03**	0.65 (0.26)	0.18 (0.40)	-72, 31
Give proposition	0.51	0.29 (0.16)	0.55 (0.79)	+89, 66	0.74	0.28 (0.16)	0.39 (0.65)	+39, 29	0.88	0.29 (0.15)	0.31 (0.55)	+6, 90	0.87	0.28 (0.14)	0.39 (0.49)	+39, 29	**0.02**	0.31 (0.15)	0.10 (0.20)	*−*67, 74	0.87	0.31 (0.14)	0.35 (0.45)	+12, 90
Give self information	0.51	0.21 (0.10)	0.10 (0.26)	*−*52, 38	0.69	0.19 (0.10)	0.13 (0.29)	*−*31, 58	0.62	0.20 (0.12)	0.35 (0.45)	+75, 00	0.87	0.18 (0.09)	0.22 (0.44)	+22, 22	0.20	0.21 (0.09)	0.43 (0.40)	+104, 76	0.74	0.21 (0.06)	0.36 (0.50)	+71, 43
Give task information	0.89	0.19 (0.18)	0.24 (0.53)	+26, 32	0.75	0.24 (0.22)	0.19 (0.38)	*−*20, 83	0.88	0.26 (0.23)	0.24 (0.51)	*−*7, 69	0.85	0.28 (0.22)	0.50 (0.71)	+78, 57	0.91	0.24 (0.23)	0.27 (0.46)	+12, 50	0.87	0.25 (0.21)	0.36 (0.81)	+44, 00
Manage task	0.89	0.28 (0.17)	0.33 (0.77)	+17, 86	0.69	0.28 (0.16)	0.40 (0.59)	+42, 86	0.69	0.30 (0.15)	0.20 (0.36)	*−*33, 33	0.85	0.28 (0.12)	0.44 (0.53)	+57, 14	0.79	0.25 (0.13)	0.36 (0.43)	+44, 00	**0.03**	0.24 (0.13)	0.05 (0.15)	*−*79, 17
Relax atmosphere	0.51	0.26 (0.16)	0.12 (0.27)	*−*53, 85	0.23	0.24 (0.14)	0.11 (0.26)	*−*54, 17	0.39	0.25 (0.13)	0.54 (0.66)	+116, 00	0.85	0.23 (0.13)	0.11 (0.33)	*−*52, 17	0.91	0.25 (0.17)	0.21 (0.26)	*−*16, 00	0.87	0.25 (0.16)	0.36 (0.67)	+44, 00
Display reflection	0.89	0.34 (0.24)	0.37 (0.47)	+8, 82	0.88	0.36 (0.28)	0.38 (0.43)	+5, 56	0.78	0.32 (0.21)	0.23 (0.40)	*−*28, 13	0.85	0.44 (0.31)	0.17 (0.50)	*−*61, 36	0.91	0.38 (0.26)	0.40 (0.53)	+5, 26	0.74	0.41 (0.30)	0.77 (0.93)	+87, 80
Manage tool	0.89	0.30 (0.23)	0.26 (0.40)	*−*13, 33	0.69	0.26 (0.20)	0.16 (0.29)	*−*38, 46	0.88	0.28 (0.20)	0.26 (0.35)	*−*7, 14	1.00	0.27 (0.20)	0.28 (0.67)	+3, 70	0.91	0.30 (0.24)	0.33 (0.47)	+10, 00	0.87	0.32 (0.23)	0.27 (0.65)	*−*15, 63

*The *p*-values are corrected for multiple comparisons with the Benjamini–Hochberg procedure. Significant results are presented in bold. M*−*, $\bar{\text{RTU}}$ when no-emotion sharing; M+, $\bar{\text{RTU}}$ when emotion sharing.*

**TABLE 4:** Comprehensive results for the receiver for each couple of shared emotion and collaborative act.

Collaborative acts	Interested (*N* = 19)				Focused (*N* = 21)				Amused (*N* = 18)				Relaxed (*N* = 12)				Satisfied (*N* = 16)				Delighted (*N* = 11)			
	*p*	*M−* (SD)	*M*+ (SD)	Variation *%*	*p*	*M−* (SD)	*M*+ (SD)	Variation *%*	*p*	*M−* (SD)	*M*+ (SD)	Variation *%*	*p*	*M−* (SD)	*M*+ (SD)	Variation *%*	*p*	*M−* (SD)	*M*+ (SD)	Variation *%*	*p*	*M−* (SD)	*M*+ (SD)	Variation *%*
Agree	0.92	0.51 (0.24)	0.50 (0.61)	*−*1, 96	0.97	0.49 (0.23)	0.48 (0.72)	*−*2, 04	0.77	0.50 (0.24)	0.38 (0.48)	*−*24, 00	0.77	0.47 (0.12)	0.29 (0.54)	*−*38, 30	0.43	0.49 (0.26)	0.32 (0.33)	*−*34, 69	0.67	0.44 (0.09)	0.64 (0.67)	+45, 45
Accept coordination	0.62	0.40 (0.23)	0.59 (0.58)	+47, 50	0.70	0.43 (0.24)	0.33 (0.41)	*−*23, 26	0.95	0.46 (0.23)	0.44 (0.82)	*−*4, 35	0.95	0.40 (0.20)	0.42 (0.47)	+5, 00	0.85	0.36 (0.13)	0.30 (0.52)	*−*16, 67	0.67	0.35 (0.17)	0.45 (0.69)	+28, 57
Give positive opinion	0.62	0.66 (0.27)	0.39 (0.78)	*−*40, 91	0.79	0.64 (0.23)	0.77 (0.86)	+20, 31	**0.03**	0.68 (0.24)	0.29 (0.44)	*−*57, 35	**0.03**	0.71 (0.24)	0.63 (0.88)	*−*11, 27	0.43	0.63 (0.24)	0.90 (0.73)	+42, 86	0.67	0.64 (0.27)	0.50 (0.74)	*−*21, 88
Give proposition	0.75	0.30 (0.17)	0.23 (0.36)	*−*23, 33	0.59	0.28 (0.16)	0.60 (1.03)	+114, 29	0.77	0.31 (0.18)	0.24 (0.39)	*−*22, 58	0.77	0.32 (0.18)	0.33 (0.44)	+3, 13	0.17	0.34 (0.15)	0.18 (0.26)	*−*47, 06	0.67	0.29 (0.11)	0.36 (0.50)	+24, 14
Give self information	0.78	0.21 (0.11)	0.17 (0.29)	*−*19, 05	0.92	0.21 (0.11)	0.23 (0.41)	+9, 52	0.77	0.21 (0.11)	0.13 (0.27)	*−*38, 10	0.77	0.17 (0.10)	0.29 (0.62)	+70, 59	0.17	0.23 (0.13)	0.09 (0.21)	*−*60, 87	0.67	0.24 (0.13)	0.18 (0.40)	*−*25, 00
Give task information	0.75	0.21 (0.20)	0.32 (0.55)	+52, 38	0.70	0.22 (0.20)	0.41 (0.74)	+86, 36	0.77	0.24 (0.21)	0.37 (0.66)	+54, 17	0.77	0.22 (0.22)	0.00 (0.00)	*−*100, 00	0.58	0.27 (0.26)	0.16 (0.32)	*−*40, 74	0.67	0.32 (0.23)	0.18 (0.40)	*−*43, 75
Manage task	0.37	0.28 (0.17)	0.12 (0.27)	*−*57, 14	**0.00**	0.30 (0.15)	0.06 (0.16)	*−*80, 00	0.95	0.27 (0.16)	0.28 (0.40)	+3, 70	0.95	0.31 (0.15)	0.21 (0.40)	*−*32, 26	0.85	0.28 (0.15)	0.32 (0.45)	+14, 29	0.33	0.28 (0.14)	0.09 (0.30)	*−*67, 86
Relax atmosphere	0.74	0.24 (0.14)	0.39 (0.57)	+62, 50	0.92	0.26 (0.14)	0.29 (0.41)	+11, 54	0.77	0.25 (0.15)	0.44 (0.56)	+76, 00	0.77	0.26 (0.17)	0.08 (0.29)	*−*69, 23	0.72	0.23 (0.16)	0.33 (0.45)	+43, 48	0.67	0.22 (0.13)	0.36 (0.50)	+63, 64
Display reflection	0.78	0.33 (0.26)	0.39 (0.62)	+18, 18	0.79	0.30 (0.21)	0.25 (0.33)	*−*16, 67	0.77	0.34 (0.29)	0.53 (0.96)	+55, 88	0.77	0.38 (0.29)	0.29 (0.40)	*−*23, 68	0.72	0.37 (0.28)	0.49 (0.66)	+32, 43	**0.02**	0.42 (0.35)	0.05 (0.15)	*−*88, 10
Manage tool	0.78	0.29 (0.23)	0.25 (0.45)	*−*13, 79	**0.00**	0.31 (0.24)	0.03 (0.11)	*−*90, 32	0.95	0.25 (0.21)	0.28 (0.54)	+12, 00	0.95	0.23 (0.19)	0.42 (0.63)	+82, 61	0.89	0.30 (0.24)	0.28 (0.45)	*−*6, 67	0.67	0.33 (0.23)	0.64 (1.21)	+93, 94

*The *p*-values are corrected for multiple comparisons with the Benjamini–Hochberg procedure. Significant results are presented in bold. M*−*, $\bar{R\,T\,U}$ when no-emotion sharing; M+, $\bar{R\,T\,U}$ when emotion sharing.*

#### Interested

The sharing of *interested* occurred mostly during phases 1 (generation of ideas) and 2 (debate about best ideas). Major increases in the $\bar{\text{RTU}}\,\text{s}$ occuring just before the emitter’s sharing concern *give proposition* (from 0.29 to 0.55; +89%). Major decreases concern *relax atmosphere* (from 0.26 to 0.12; −53%), *give self information* (from 0.21 to 0.10; −52%), and *accept coordination* (from 0.46 to 0.31; −32%). Major increases in the $\bar{\text{RTU}}\,\text{s}$ of the receiver occur just after the emitter’s sharing concern *relax atmosphere* (from 0.24 to 0.39; +62%), *give task information* (from 0.21 to 0.32; +52%), and *accept coordination* (from 0.40 to 0.59; +47%). Major decreases concern *manage task* (from 0.28 to 0.12; −57%) and *give positive opinion* (from 0.66 to 0.39; −41%).

#### Focused

The sharing of *focused* occurred mostly during phases 1 and 2. Major increases in the $\bar{\text{RTU}}\,\text{s}$ occuring just before the emitter’s sharing concern *manage task* (from 0.28 to 0.40; +42%) and *give proposition* (from 0.28 to 0.39; +39%). Major decreases concern *relax atmosphere* (from 0.24 to 0.11; −54%), *give positive opinion* (from 0.69 to 0.32; −53%), and *manage tool* (from 0.26 to 0.16; −38%). Major increases in the $\bar{\text{RTU}}\,\text{s}$ of the receiver occuring just after the emitter’s sharing concern *give proposition* (from 0.28 to 0.60; +114%) and *give task information* (from 0.22 to 0.41; +86%). Major decreases concern *manage tool* (from 0.31 to 0.03; −90%) and *manage task* (from 0.30 to 0.06; −80%). A significant decrease in the $\bar{\text{RTU}}\,\text{s}$ of the emitter was found for the collaborative act *give positive opinion* (*p* = 0.02). For the receiver, significant decreases were found for the collaborative acts *manage task* (*p* < 0.001) and *manage tool* (*p* < 0.001).

#### Amused

The sharing of *amused* occurred mostly during phase 1. Major increases in the $\bar{\text{RTU}}\,\text{s}$ occuring just before the emitter’s sharing concern *relax atmosphere* (from 0.25 to 0.54; +116%), *give self information* (from 0.20 to 0.35; +75%), and *agree* (from 0.46 to 0.61; +32%). Major decreases concern *accept* (from 0.42 to 0.22; −47%) and *manage task* (from 0.30 to 0.20; −33%). Major increases in the $\bar{\text{RTU}}\,\text{s}$ of the receiver occuring just after the emitter’s sharing concern *relax atmosphere* (from 0.25 to 0.44; +76%), *display reflection* (from 0.34 to 0.53; +55%), and *give task information* (from 0.24 to 0.37; +54%). Major decreases concern *give positive opinion* (from 0.68 to 0.29; −57%) and *give self information* (from 0.21 to 0.13; −38%). A significant decrease in the $\bar{\text{RTU}}\,\text{s}$ of the receiver was found for the collaborative act *give positive opinion* (*p* = 0.03).

#### Relaxed

The sharing of *relaxed* occurred in roughly equivalent proportions across phases. Major increases in the $\bar{\text{RTU}}\,\text{s}$ occuring just before the emitter’s sharing concern *give task information* (from 0.28 to 0.50; +78%), *accept coordination* (from 0.34 to 0.56; +64%), *manage task* (from 0.28 to 0.44; +57%), and *give proposition* (from 0.28 to 0.39; +39%). Major decreases concern *display reflection* (from 0.44 to 0.17; −61%) and *relax atmosphere* (from 0.23 to 0.11; −52%). Major increases in the $\bar{\text{RTU}}\,\text{s}$ of the receiver occuring just after the emitter’s sharing concern *manage tool* (from 0.23 to 0.42; +82%) and *give self information* (from 0.17 to 0.29; +70%). Major decreases concern *give task information* (from 0.22 to 0; −100%), *relax atmosphere* (from 0.26 to 0.08; −69%), *agree* (from 0.47 to 0.29; −38%), and *manage task* (from 0.31 to 0.21; −32%). A significant decrease in the $\bar{\text{RTU}}\,\text{s}$ of the receiver was found for the collaborative act *give positive opinion* (from 0.71 to 0.63; −11%; *p* = 0.03).

#### Satisfied

The sharing of *satisfied* occurred in roughly equivalent proportions in each phase, with a slight increase across phases. Major increases in the $\bar{\text{RTU}}\,\text{s}$ occuring just before the emitter’s sharing concern *give self information* (from 0.21 to 0.43; +104%) and *manage task* (from 0.25 to 0.36; +44%). Major decreases concern *give proposition* (from 0.31 to 0.10; −67%) and *accept coordination* (from 0.41 to 0.22; −46%). Major increases in the $\bar{\text{RTU}}\,\text{s}$ of the receiver occuring just after the emitter’s sharing concern *relax atmosphere* (from 0.23 to 0.33; +43%), *give positive opinion* (from 0.63 to 0.90; +42%), and *display reflection* (from 0.37 to 0.49; +32%). Major decreases concern *give self information* (from 0.23 to 0.09; −60%), *give proposition* (from 0.34 to 0.18; −47%), and *give task information* (from 0.27 to 0.16; −40%). A significant decrease in the $\bar{\text{RTU}}\,\text{s}$ of the receiver was found for the collaborative act *give proposition* (*p* = 0.02).

#### Delighted

The sharing of *delighted* occurred mostly during phases 1 and 3 (choose final idea). Major increases in the $\bar{\text{RTU}}\,\text{s}$ occuring just before the emitter’s sharing concern *display reflection* (from 0.41 to 0.77; +87%), *give self information* (from 0.21 to 0.36; +71%), *give task information* (from 0.25 to 0.36; +44%), and *relax atmosphere* (from 0.25 to 0.36; +44%). Major decreases concern *manage task* (from 0.24 to 0.05; −79%), *give positive opinion* (from 0.65 to 0.18; −72%), and *accept coordination* (from 0.43 to 0.24; −44%). Major increases in the $\bar{\text{RTU}}\,\text{s}$ of the receiver occuring just after the emitter’s sharing concern *manage tool* (from 0.33 to 0.64; +93%), *relax atmosphere* (from 0.22 to 0.36; +63%), and *agree* (from 0.44 to 0.64; +45%). Major decreases concern *display reflection* (from 0.42 to 0.05; −88%), *manage task* (from 0.28 to 0.09; −67%), and *give task information* (from 0.32 to 0.18; −43%). Significant decreases in the $\bar{\text{RTU}}\,\text{s}$ of the emitter were found for the collaborative acts *give positive opinion* (*p* = 0.03) and *manage task* (*p* = 0.03). A significant decrease in the $\bar{\text{RTU}}\,\text{s}$ of the receiver was found for the collaborative act *display reflection* (*p* = 0.02).

## Discussion

### Does Emotion Sharing Modulate Collaborative Acts?

The first question that drove this study concerned a potential effect of emotional sharing on collaborative acts (RQ1). We proposed above that, in collaborative settings, the expression of emotion by collaborators could, at specific points of time, (1) from the emitter’s point of view, help to draw the other’s attention implicitly and explicitly on socio-relational and socio-epistemic matters and (2) from the receiver’s point of view, focus on the other’s emotional state to make inferences, reduce ambiguity, and adapt to the emitter’s needs. Drawing on the literature, we proposed that real-time changes of the emitter’s collaborative acts occur before the emitter’s emotional sharings (H1a) and real-time changes of the receiver’s collaborative acts follow the emitter’s emotional sharing (H1b).

Some effects of emotional sharing have been found in both directions. First, emotional sharing does relate to how emitters use some collaborative acts just before. Indeed some significant variations in the sharing of some emotions occur only before some emotional sharing and do not occur without emotion sharing. Accordingly, emotional sharing does have an impact on how receivers use collaborative acts just after. Indeed some significant variations in the receiver’s collaborative acts occur only after the sharing of some emotions by the emitter. These results confirm that emotional sharing modulates collaborative acts in problem-solving and strongly suggest that emotional sharing is probably a way for the emitter to highlight his/her previous collaborative acts to elicit adaptive changes from the receiver.

However, a surprising result is that emotional sharing only induced significant $\bar{\text{RTU}}\,\text{s}$ decreases. In a window of *n* collaborative acts, decreases in some collaborative acts are compensated by increases in some others. However, in our case, if some decreases reached the significance level, it was not the case for the increases. In other words, if emotional sharing did produce increases in some collaborative acts compensating the decrease in some others, these increases were not consistent enough among the whole sample. How can one explain that emotional sharing only produces consistent decreases in collaborative acts? We propose two different explanations that can complement each other. First, emotional sharing would put on hold the ongoing dynamics of collaborative problem-solving. In other words, the emitter would stop to share collaborative acts related to the ongoing matter to promote a change from the receiver through emotion sharing. As this message has to be understood by the receiver to adjust his/her subsequent collaborative acts, it would also cut the receiver’s collaborative acts dedicated to the ongoing matter. If this process would consistently decrease some collaborative acts, it could also lead to some discrepancies between participants, some of them adjusting the collaborative acts more or less rapidly. Second, the receivers would react differently and quite inconsistently to emotional sharing. In other words, receivers would implement different ways to answer the emotional message coming from the emitter. Therefore, an increase in a given collaborative act would be less likely to reach the significance level across the whole sample studied.

### Are There Patterns of Collaboration Related to Some Emotion Sharings?

The second question that drove this study was whether some patterns of collaborative acts involving specific emotional sharings can be found (RQ2). We proposed that specific triads relate more specifically to dealing with specific epistemic or relational matters (H2a). We also assumed that some triads occur preferentially in specific collaboration phases (H2b). In the discussion below, we will assume the role of emotion sharing in considering both the significant results when available and the major $\bar{\text{RTU}}$ variations described in the descriptive results. Supplementary Appendix B presents the examples taken from the conversation for an illustrative purpose.

The sharing of *interested* would primarily occur when the participants produce and debate ideas to solve the problem. At the epistemic level, sharing *interested* could follow the willingness to draw the partner’s attention to the fact that a previous contribution could provide a line of thought in a moment of brainstorming, where the emitter mainly generates new ideas. The collaborative partner would respond to this interest by mainly accepting more coordination, reminding more the task rules, and managing less the progress of the task. In the relational space, the emitter would alleviate less the atmosphere before emitting *interest.* This could be congruent with the assumption that socio-cognitive tensions may emerge from the search of ideas (Andriessen et al., 2011), perhaps starting sometime before the sharing of *interest*. In response, collaborative acts dedicated to alleviating this tension (Andriessen et al., 2011) would be emitted by the receiver since a potential solution has just emerged. All in all, sharing *interest* would mark a pivotal point between the search and the discovery of a good idea to solve the problem at the epistemic level, which would go hand in hand with a transition between socio-cognitive tension and relaxation at the relational level.

The sharing of *focused* would also primarily occur when the participants produce and debate ideas to solve the problem. At the epistemic level, contrary to the sharing of *interest*, *focused* seems to draw the receiver’s attention to the fact that the previous contributions are still not satisfying, and further thinking is needed. The collaborative partner would start, in turn, to generate new ideas and decrease managing the task and using the collaborative tool. At the relational level, contrary to the sharing of *interest*, the sharing of *focused* would not lead to alleviating the socio-cognitive tension as no good idea has emerged yet. All in all, sharing *focused* would be intended to increase group thinking in the search for a good idea.

The sharing of *amused* would primarily occur when the participants produce ideas. Sharing *amused* could be a way to highlight the desire to strengthen group harmony at both relational and epistemic levels. At the epistemic level, the emitter would agree more on new ideas and share more information about his/her thinking. Instead he/she would accept less group coordination and manage less the task progress. In return, the emitter would display more reflection and give more task information. At the relational level, both partners would improve the atmosphere when *amused* is shared. Thus, the sharing of *amused* could mark quick consensus building, which refers to a rapid agreement of the other’s contribution, aiming at promoting the generation of ideas instead of confronting them (Weinberger and Fischer, 2006), which goes hand in hand with an attempt to improve the group atmosphere.

The sharing of *relaxed* would occur in roughly equivalent proportions across phases. At the cognitive level, it would mark a phase mixing the search of new ideas with the task, group, and tool management. The participants could give free rein to their ideas. At the relational level, contributions dedicated to alleviating the atmosphere would also decrease in both sides, possibly marking a phase of socio-cognitive relaxation.

The sharing of *satisfied* would also occur in roughly equivalent proportions across phases. At the epistemic level, the emitter would signal that a satisfactory proposition is found in giving significantly less new ideas just before. He/she would also give more information about his/her own thinking, perhaps for explaining his/her view. The receiver would accept less group coordination and give fewer new ideas. Instead he/she would mainly give more positive opinions about previous ideas and more information about his/her own thinking. At the relational level, the sharing of *satisfied* would alleviate tension in the group as collaborative acts dedicated to improving the atmosphere increase.

Finally, the sharing of *delighted* would primarily occur when the participants produce ideas and choose the best idea in the end. At the epistemic level, *delighted* would mark a transition between successful collaborative work and the beginning of a moment dedicated to the management of the collaborative tool. On the one hand, the emitter would decrease the management of the task progress, display more reflection and information about his/her thinking, and produce fewer positive opinions about previous ideas. On the other hand, the receiver would agree with previous ideas and manage the tool more, probably for writing down the solution(s) found. He/she would also decrease reflection, perhaps adapting him/herself to an increase of reflection from the emitter, and decrease the management of the task progress. At the relational level, the sharing of *delighted* would occur in a relaxation phase as both emitter and receiver would produce more collaborative acts dedicated to relaxing the atmosphere. The sharing of *delighted* and *satisfied* seems to be used in a quite similar fashion. However, delighted would mark more definitive solutions.

The preliminary results obtained strongly suggest that emotional sharing intervenes in different aspects and phases of collaborative problem-solving. However, emotional sharing does not appear to relate solely to epistemic and relational matters, and relational and epistemic spaces appear to be closely intertwined.

### Limitations

As with any research involving naturalistic collaboration settings, the present study is not without limitations. First, our research highlights the difficulty of studying quantitatively emotional sharing because it is challenging to make the participants share emotions “on demand.” Explicit emotional sharing appears to be a discrete process that responds to specific problem-solving situations. These kinds of situations appear at some specific moments and can hardly be experimentally scheduled. As a consequence, the number of occurrences of each emotion of interest appeared relatively small (about two, on average, for a 20-min collaborative exchange). Therefore, higher variability in the $\bar{\text{RTU}}\,\text{s}$ of the emotion windows produced less robust changes across the participants than in the no-emotion windows. This could also partly explain why collaborative act increases failed to reach significance. For this reason, we are deeply aware that the results highlighted in this article, although they confirm the role of emotion sharing and give a first insight of the interrelations that may exist between emotion sharing and collaborative acts, need to be deepened and replicated.

A second related issue that results from the difficulty of manipulating emotional sharing was the very small number of some emotions in the sample, which prevented us from including them in the current analysis. Especially the potential effects of negative emotions on the partner’s collaborative acts have been completely overlooked. In a previous analysis conducted on the same sample (Avry et al., 2015), the researchers analyzed the difference between the proportion of emotions experienced by problem-solvers vs. the proportion of emotions shared. If the participants shared more positive emotions as they really experienced (86 vs. 71%), highlighting that the sharing of positive emotions could play an instrumental role in problem-solving regulation, the reverse pattern was found for negative emotions. Indeed the participants experienced twice as many negative emotions as they share (28.7 vs. 14%). This result strongly suggests that the participants refrain themselves to share explicitly negative emotions in collaboration settings, potentially to prevent unwanted negative impact on the group. However, there is a strong likelihood that the sharing of negative affects would also induce similar or even more potent effects on the partner’s collaborative acts, but perhaps more implicitly (e.g., through para- and non-verbal communication). A way to increase the sharing of some emotions (and especially negative emotions) without affecting the naturalistic characteristics of collaboration could be to design more constrained collaborative situations. For example, a situation where reaching a joint agreement would be impossible could generate specific negative emotions (e.g., frustration and confusion). Furthermore, analyzing larger samples will also increase the overall reliability of the results obtained.

## Conclusion and Perspective

This study was conducted as a premise to a more global and deeper comprehension of the dynamics between emotional sharing and communicative exchanges in collaborative problem-solving. First, we examined if real-time adaptive changes in the emitter’s collaborative acts influence his/her emotional sharing and if the emitter’s emotional sharing induces real-time adaptive changes in the receiver’s collaborative acts. Second, we investigated if some specific patterns of the emitter’s collaborative acts, the emitter’s emotional sharings, and the receiver’s collaborative acts have privileged relationships in the different phases of the collaboration. First, we confirmed that emotional sharing follows and induces a rapid modulation of the emitter’s and the receiver’s collaborative acts. This result fits with the idea that emotional sharing regulates collaborative problem-solving in the same way that it regulates social interaction more broadly. As proposed by some researchers (Keltner and Haidt, 1999; Van Doorn et al., 2015; Van Kleef and Fischer, 2016; Van Kleef et al., 2016) regarding social interaction, the emitter’s emotional expressions may be used to draw the partner’s attention and elicit adaptive changes regarding socio-relational and socio-cognitive matters. Meanwhile, the partner needs to infer the emitter’s beliefs, intentions, and orientations regarding both the cognitive (i.e., how the problem is solved) and the relational space (i.e., how the group interacts) in the context of collaborative problem-solving. In addition, we highlighted that specific patterns of emotion sharings and collaborative acts relate more specifically to dealing with specific matters in the different phases of the collaboration. These findings also suggest that emotion sharing would initiate different collaborative epistemic stages. These epistemic changes would also involve concomitant relational changes, especially the modulation of tensions and relaxations in the group. Furthermore, even if this assumption has to be confirmed, our results suggest that the receivers would adapt their collaborative acts in different ways for a given emotional sharing. Therefore, other factors have to be explored (e.g., history of the dyadic relationship, beliefs about competence, and motivational aspects) to understand more finely the dynamics between the emitter’s and the receiver’s collaborative acts.

By and large, this work highlights the value of studying collaborative problem-solving with the emotional aspects that pervade it. Based on these preliminary findings, some perspectives can be considered in both educational psychology and computer science fields. First, if emotional sharing leads to the modulation of subsequent collaborative acts, it could be promoted as a way to understand and leverage group reflection, decisions, and actions in collaborative complex learning such as problem-solving. In this way, it could be particularly useful for promoting emotional regulation and strengthening emotional competencies among problem-solvers (Mayer et al., 2000; Mikolajczak et al., 2014) as a socio-metacognitive and meta-relational artifact (Hogan, 1999). Second, in combination with natural language processing (semantic analysis of speech utterances; Baets et al., 2019) and process mining (analysis of patterns of collaborative acts; Van der Aalst, 2011) techniques, emotional sharing data could help to gain a better insight into the bottlenecks as well as the facilitators of successful collaborative problem-solving.

## Data Availability Statement

The datasets generated for this study are available on request to the corresponding author.

## Ethics Statement

The studies involving human participants were reviewed and approved by the ethics committee of the Faculty of Psychology and Educational Science of the University of Geneva. The patients/participants provided their written informed consent to participate in this study.

## Author Contributions

SA: conceptualization, methodology, software, formal analysis, investigation, resources, and writing – original draft and visualization. GM and MB: conceptualization. GC: conceptualization, methodology, software, investigation, and writing – review and editing.

## Conflict of Interest

The authors declare that the research was conducted in the absence of any commercial or financial relationships that could be construed as a potential conflict of interest.

## Footnotes

1 https://eatmint.unige.ch/

## References

[B1] Andriessen J. Baker M. van der Puil C. (2011). Socio-cognitive tension in collaborative working relations. *Learn. Across Sites New Tools Infrastru. Pract.* 8 222–242.

[B2] Austin J. L. (1975). *How To Do Things With Words.* Oxford: Oxford University Press.

[B3] Avry S. Chanel G. Bétrancourt M. Molinari G. (2020). Achievement appraisals, emotions and socio-cognitive processes: how they interplay in collaborative problem-solving? *Comput. Hum. Behav.* 107:106267 10.1016/J.CHB.2020.106267

[B4] Avry S. Molinari G. Chanel G. Betrancourt M. Pun T. (2015). *The Display (Or Masking) Of Emotions During Computer-Mediated Interaction: A Relationship With Reappraisal.* Genève: International Society for Research on Emotion.

[B5] Baets W. Oldenboom E. Hosken C. (2019). *The Potential of Semantic Analysis for Business (Education).* 10.2139/ssrn.3364133

[B6] Baker M. Andriessen J. Lund K. van Amelsvoort M. Quignard M. (2007). Rainbow: a framework for analysing computer-mediated pedagogical debates. *Intern. J. Comput. Support. Collab. Learn.* 2 315–357. 10.1007/s11412-007-9022-4

[B7] Bales R. F. (1950). A set of categories for the analysis of small group interaction. *Am. Sociol. Rev.* 15 257–263.

[B8] Barron B. (2003). When smart groups fail. *J. Learn. Sci.* 12 307–359. 10.1207/s15327809jls1203_1

[B9] Borge M. Aldemir T. Xia Y. (2019). “Unpacking socio-metacognitive sense-making patterns to support collaborative discourse,” in *Proceedings of the Computer Supported Collaborative Learning (CSCL)*, Lyon.

[B10] Borge M. Ong Y. S. Rosé C. P. (2018). Learning to monitor and regulate collective thinking processes. *Intern. J. Comput. Support. Collab. Learn.* 13 61–92. 10.1007/s11412-018-9270-5

[B11] Chanel G. Betrancourt M. Pun T. Cereghetti D. Molinari G. (2013). “Assessment of computer-supported collaborative processes using interpersonal physiological and eye-movement coupling,” in *Proceedings of the Affective Computing and Intelligent Interaction (ACII), 2013 Humaine Association Conference*, (Piscataway, NJ), 116–122. 10.1109/ACII.2013.26

[B12] Dillenbourg P. Lemaignan S. Sangin M. Nova N. Molinari G. (2016). The symmetry of partner modelling. *Intern. J. Comput. Support. Collab. Learn.* 11 227–253. 10.1007/s11412-016-9235-5

[B13] D’Mello S. Graesser A. (2012). Dynamics of affective states during complex learning. *Learn. Instruct.* 22 145–157. 10.1016/j.learninstruc.2011.10.001

[B14] Eastwood J. D. Smilek D. Merikle P. M. (2001). Differential attentional guidance by unattended faces expressing positive and negative emotion. *Percept. Psychophys.* 63 1004–1013. 10.3758/bf03194519 11578045

[B15] Efklides A. (2011). Interactions of metacognition with motivation and affect in self-regulated learning: the MASRL model. *Educ. Psychol.* 46 6–25. 10.1080/00461520.2011.538645

[B16] Fiore S. M. Graesser A. Greiff S. Griffin P. Gong B. Kyllonen P. (2017). *Collaborative Problem Solving: Considerations For The National Assessment Of Educational Progress.* Available online at: https://nces.ed.gov/nationsreportcard/pdf/researchcenter/collaborative_problem_solving.pdf

[B17] Fischer A. H. Manstead A. S. (2008). Social functions of emotion. *Handb. Emot.* 3 456–468.

[B18] Fredrickson B. L. (2013). Positive emotions broaden and build. *Adv. Exper. Soc. Psychol.* 47 1–53. 10.1016/b978-0-12-407236-7.00001-2

[B19] George J. M. Dane E. (2016). Affect, emotion, and decision making. *Organ. Behav. Hum. Decision Process.* 136 47–55. 10.1016/j.obhdp.2016.06.004

[B20] Graesser A. C. Fiore S. M. Greiff S. Andrews-Todd J. Foltz P. W. Hesse F. W. (2018). Advancing the science of collaborative problem solving. *Psychol. Sci. Public Interest* 19 59–92. 30497346 10.1177/1529100618808244

[B21] Hogan K. (1999). Sociocognitive roles in science group discourse. *Intern. J. Sci. Educ.* 21 855–882. 10.1080/095006999290336

[B22] Hughes M. Susie V. Mark D. (2007). Assessing social presence in online discussion groups: a replication study. *Innovat. Educ. Teach. Intern.* 44 17–29. 10.1080/14703290601090366

[B23] Huntsinger J. R. Schnall S. (2013). *Emotion–Cognition Interactions.* Oxford: Oxford University Press.

[B24] Isen A. M. Labroo A. A. (2012). “Some ways in which positive Affect facilitates decision making and judgment,” in *Emerging Perspectives On Judgment And Decision Research*, eds Schneider S. L. Shanteau J. (New York, NY: The Guilford Press), 365–393. 10.1017/cbo9780511609978.013

[B25] Jaillon P. Corbel A. Girardot J. (2002). “DREW: a dialogical reasoning web tool,” in *Proceedings of the ICTE2002, International Conference on ICT’s in Education*, Badajoz.

[B26] Kahrimanis G. Meier A. Chounta I. A. Voyiatzaki E. Spada H. Rummel N. (2009). “Assessing collaboration quality in synchronous CSCL problem-solving activities: adaptation and empirical evaluation of a rating scheme,” in *Proceedings of the European Conference on Technology Enhanced Learning*, (Berlin: Springer), 267–272. 10.1007/978-3-642-04636-0_25

[B27] Keltner D. Haidt J. (1999). Social functions of emotions at four levels of analysis. *Cogn. Emot.* 13 505–521. 10.1080/026999399379168

[B28] Lerner J. S. Li Y. Valdesolo P. Kassam K. S. (2015). Emotion and decision making. *Annu. Rev. Psychol.* 45 133–155. 10.1146/annurev-psych-010213-115043 25251484

[B29] Mayer J. D. Caruso D. R. Salovey P. (2000). “Selecting a measure of emotional intelligence: the case for ability scales,” in *The Handbook Of Emotional Intelligence: Theory, Development, Assessment, And Application At Home, School, And In The Workplace*, eds Bar-On R. Parker J. D. A. (San Francisco, CA: Jossey-Bass), 320–342.

[B30] Meier A. Spada H. Rummel N. (2007). A rating scheme for assessing the quality of computer-supported collaboration processes. *Intern. J. Comput. Support. Collab. Learn.* 2 63–86. 10.1007/s11412-006-9005-x

[B31] Mikolajczak M. Quoidbach J. Kotsou I. Nelis D. (2014). *Les Compétences Émotionnelles.* Paris: Dunod.

[B32] Molinari G. Chanel G. Bétrancourt M. Pun T. Bozelle Giroud C. (2013). “Emotion feedback during computer-mediated collaboration: Effects on self-reported emotions and perceived interaction,” in *Proceedings of the 10th International Conference on Computer-Supported Collaborative Learning (CSCL 2013)*, New York, NY.

[B33] Morris M. W. Keltner D. (2000). How emotions work: the social functions of emotional expression in negotiations. *Res. Organ. Behav.* 22 1–50. 10.1016/s0191-3085(00)22002-9

[B34] Noroozi O. Weinberger A. Biemans H. J. Teasley S. D. Mulder M. (2012). “Fostering multidisciplinary learning through computer-supported collaboration script: the role of a transactive memory script,” in *Proceedings of theEuropean Conference on Technology Enhanced Learning*, Berlin.

[B35] Ong D. C. Zaki J. Goodman N. D. (2015). Affective cognition: exploring lay theories of emotion. *Cognition* 143 141–162. 10.1016/j.cognition.2015.06.010 26160501

[B36] Pekrun R. (2006). The control-value theory of achievement emotions: assumptions, corollaries, and implications for educational research and practice. *Educ. Psychol. Rev.* 18 315–341. 10.1007/s10648-006-9029-9

[B37] Pekrun R. Linnenbrink-Garcia L. (2012). “Academic emotions and student engagement,” in *Handbook of Research On Student Engagement*, eds Christenson S. Reschly A. Wylie C. (Berlin: Springer), 259–282. 10.1007/978-1-4614-2018-7_12

[B38] Rimé B. (2007). Interpersonal emotion regulation. *Handb. Emot. Regul.* 1 466–468.

[B39] Rimé B. (2009). Emotion elicits the social sharing of emotion: theory and empirical review. *Emot. Rev.* 1 60–85. 10.1177/1754073908097189

[B40] Roschelle J. Teasley S. D. (1995). “The construction of shared knowledge in collaborative problem solving,” in *Computer Supported Collaborative Learning*, ed. O’Malley C. E. (Berlin: Springer).

[B41] Sangin M. Nova N. Molinari G. Dillenbourg P. (2007). “Partner modeling is mutual,” in *Proceedings of the 8th International Conference On Computer Supported Collaborative Learning*, (Nashville, TN: International Society of the Learning Sciences).

[B42] Sbisà M. (2009). Speech act theory. *Key Notions Pragm.* 1 229–344.

[B43] Sloetjes H. Wittenburg P. (2008). “Annotation by category-ELAN and ISO DCR,” in *Proceedings of the 6th international Conference on Language Resources and Evaluation (LREC 2008)*, Marrakesh.

[B44] Spering M. Wagener D. Funke J. (2005). The role of emotions in complex problem-solving. *Cogn. Emot.* 19 1252–1261.

[B45] Van der Aalst W. M. “Process[discovery: an introduction,” in *Process Mining*, (Berlin: Springer), 125–156. 10.1007/978-3-642-19345-3_5

[B46] Van Doorn E. A. van Kleef G. A. van der Pligt J. (2015). How emotional expressions shape prosocial behavior: interpersonal effects of anger and disappointment on compliance with requests. *Motiv. Emot.* 39 128–141. 10.1007/s11031-014-9421-6

[B47] Van Kleef G. A. Cheshin A. Fischer A. H. Schneider I. K. (2016). The social nature of emotions. *Front. Psychol.* 7:896. 10.3389/fpsyg.2016.00896 27378990 PMC4906017

[B48] Van Kleef G. A. De Dreu C. K. Manstead A. S. (2010). “An interpersonal approach to emotion in social decision making: the emotions as social information model,” in *Advances in Experimental Social Psychology*, Vol. 42 ed. Zanna M. P. (Cambridge, MA: Academic Press), 45–96. 10.1016/s0065-2601(10)42002-x

[B49] Van Kleef G. A. Fischer A. H. (2016). Emotional collectives: how groups shape emotions and emotions shape groups. *Cogn. Emot.* 30 3–19. 10.1080/02699931.2015.1081349 26391957

[B50] Viera A. J. Garrett J. M. (2005). Understanding interobserver agreement: the kappa statistic. *Fam. Med.* 37 360–363. 15883903

[B51] Weinberger A. Fischer F. (2006). A framework to analyze argumentative knowledge construction in computer-supported collaborative learning. *Comput. Educ.* 46 71–95. 10.1016/j.compedu.2005.04.003

