# Supplementary material for: Sharing Emotions Contributes to Regulating Collaborative Intentions in Group Problem-Solving
Source: Front Psychol. 2020 Jun 16;11:1160. doi: 10.3389/fpsyg.2020.01160 (PMC7308483; doi:10.3389/fpsyg.2020.01160)
Supplement: Supplementary file 1 [file Table_1.pdf]

Appendix A

Table A1

Number of collaborative acts and percentage for each category for the whole sample

Collaborative process	Collaborative act	Number of acts	Percentage relative to the total number of acts
	Display solidarity	23	0.44
Relationship management	**Relax atmosphere**	**232**	**4.51**
	Use social convention	38	0.74
	Check reception	69	1.34
	Check comprehension	16	0.31
	Display active listening	122	2.37
Interaction management	**Display reflection**	**448**	**8.71**
	Coordinate teamwork	102	1.98
	**Accept coordination**	**425**	**8.27**
	Refuse coordination	12	0.23
	**Give Task Information**	**219**	**4.26**
	Give Explanation	18	0.35
	Elicit Task Information	87	1.69
Information management	**Give Self Information**	**215**	**4.18**
	Elicit Partner Information	60	1.17
	Give recall	153	2.98
	Elicit recall	16	0.31
	**Give proposition**	**295**	**5.74**
	**Give positive opinion**	**619**	**12.04**
	Give negative opinion	62	1.21
Argumentation management	Elicit proposition	30	0.58
	Elicit opinion	132	2.56
	**Agree**	**495**	**9.63**
	Incorporate	38	0.74
Task management	**Manage task**	**310**	**6.03**
Tool management	**Manage tool**	**309**	**6.01**
Other	Other	561	10.91
Outside activity	Outside activity	34	0.61

Table A2

Number of emotions, percentage for each category and number of participants where the sharing occurred at least once for the whole sample, not including emotions removed due to overlap or absence of at least five consecutive collaborative acts before or after

Emotion	Number of sharing	Percentage relative to the total number of emotions	Number of participants where the sharing occurred at least once
**Focused**	**50**	**21.01**	**21**
**Interested**	**37**	**15.61**	**19**
**Satisfied**	**35**	**14.77**	**16**
**Amused**	**33**	**13.92**	**18**
Confident	16	6.75	9
**Relaxed**	**17**	**7.17**	**12**
**Delighted**	**12**	**5.06**	**11**
Confused	9	3.79	6
Empathic	5	2.11	4
Stressed	4	1.69	2
Annoyed	4	1.69	1
Anxious	2	0.84	2
Relieved	2	0.84	2
Dissatisfied	2	0.84	2
Bored	2	0.84	1
Frustrated	2	0.84	2
Grateful	1	0.42	1
Disappointed	1	0.42	1
Surprised	1	0.42	1
Envious	0	0	0
