# Supplementary material for: Sharing Emotions Contributes to Regulating Collaborative Intentions in Group Problem-Solving
Source: Front Psychol. 2020 Jun 16;11:1160. doi: 10.3389/fpsyg.2020.01160 (PMC7308483; doi:10.3389/fpsyg.2020.01160)
Supplement: Supplementary file 2 [file Table_2.pdf]

Appendix B

Interested

P1	Wait, let's write one slogan	Manage task
P1	We can create some messages for the agressors and some other for the victims	Give proposition
P2	*Without violence you're stronger*	Give proposition
P1	I was thinking, maybe it is not adapted to victims	Give negative opinion
P1	An eye for an eye, a tooth for a tooth ?	Give proposition
P1	For me it's the right message, but we should make it more attractive	Give proposition
P1	**Interested**	
P2	Yes, why not?	Give positive opinion
P2	It could be a way to involve victims	Give positive opinion
P1	Yes because at some point maybe the victim is too submissive	Give positive opinion
P2	Ok, let's think about that idea	Accept coordination
P1	And it is like a vicious circle	Give positive opinion
P1	Do you want to be part of a vicious circle ?	Give proposition
P2	(Chuckles)	Relax atmosphere
P2	Hit him, do not hit you ?	Give proposition

Focused

P2	We can speak together now right?	Elicit task information
P1	Yes	Give task information
P1	*You could be the victim*	Give proposition
P1	*Strike with your arguments*	Give proposition
P2	You ruin your life and everyone else's ?	Give proposition
P1	It could be fun to have a slogan in english	Give proposition
P2	I'm not very good in English but we can try yes	Agree
P1	Ok, no problem	Accept coordination
P1	**Focused**	
P2	Hmm	Show reflection
P2	Do unto others as you would have others do unto you ?	Give proposition
P2	Punches are for cowards, be clever ?	Give proposition
P2	Hmm	Show reflection
P1	It could be nice to make a pun	Give proposition
P2	Oh, we could look for our slogan with a word battle	Manage task
P1	Yes why not ?	Manage task
P2	How can we do that ?	Coordinate teamwork

Amused

P1	(Chuckles)	Relax atmosphere
P1	It is not easy !	Give self information
P1	Can we start to make links between ideas at this step?	Elicit task information
P2	Yes I think so	Give task information
P1	This one is very funny (Chuckles)	Relax atmosphere
P1	*Violence, addiction of feeble-minded*, well done!	Show solidarity
P2	(Chuckles) yes, thank you	Relax atmosphere
P1	**Amused**	
P2	Hmm	Display reflection
P1	We could say that violence is like an addiction ?	Give proposition
P2	Yes, exactly	Agree
P2	(Chuckles)	Relax atmosphere
P2	They do not have the power of ideas but the power of muscles	Give explanation
P1	(Chuckles) True !	Relax atmosphere
P2	(Chuckles)	Relax atmosphere

Relaxed

P1	We could add another slogan	Manage task
P1	*You are not a punchball*	Give proposition
P2	(Chuckles)	Relax atmosphere
P1	I don't know, I am not convinced by this one	Give negative opinion
P2	Let's put our random ideas here	Coordinate teamwork
P1	Ok	Accept coordination
P1	I think we should add the idea that cool people are not violent	Give proposition
P1	**Relaxed**	
P2	Ok let's write down this idea	Tool discourse
P2	May we add something else	Tool discourse
P1	Is that cool to pull fly wings out ?	Give proposition
P1	It has just come to my mind	Give self information
P2	I have some difficulty to imagine the message that would make a difference	Give self information
P1	Hmm	Show reflection
P2	Otherwise I have another idea	Give self information
P2	Remember those who died before hitting	Give proposition

Satisfied

P1	Phase n°2 is done, isn't it?	Manage task
P2	Yes	Manage task
P1	For me, this one is among the finalists	Manage task
P2	Yes, I'm ok with you	Manage task
P1	It's a good mix between both our ideas	Give positive opinion
P2	Yes exactly !	Agree
P1	A beautiful mixed-breed (Chuckles)	Relax atmosphere
P2	I'm quite satisfied !	Give self information
P1	Me too !	Give self information
P1	**Satisfied**	
P2	We can now submit it to the city of Geneva (Chuckles)	Relax atmosphere
P1	(Chuckles) I don't know if they wish to use it to be honest	Relax atmosphere
P1	We never know, we never know	Relax atmosphere
P2	It is good yes !	Give positive opinion
P2	But I don't think so (Chuckles)	Relax atmosphere
P1	(Chuckles)	Relax atmosphere
P2	Hmm	Show reflection
P2	So, how long is expected to last Phase n°3 ?	Elicit task information

Delighted

P1	I find it original	Give self information
P1	I've tried to make words rhyme	Give self information
P1	*You are the boss of your classroom but if it were you the scapegoat?*	Give recall
P2	But uh…	Display reflection
P2	Ok, it works !	Give positive opinion
P1	Ok	Agree
P2	Uh…	Display reflection
P1	So, we have to write in the tool now	Give task information
P1	**Delighted**	
P2	Original according to the rhyme, right?	Elicit opinion
P1	Yes	Agree
P2	Ok, I write rhyme here	Manage Tool
P2	And I move that below	Manage Tool
P1	Ok	Manage Tool
P2	Do I link it with appropriate also?	Manage Tool
P1	Yes, I think so	Manage Tool
